# Maintenance regimen of GM‐CSF with rituximab and lenalidomide improves survival in high‐risk B‐cell lymphoma by modulating natural killer cells

**DOI:** 10.1002/cam4.5969

**Published:** 2023-04-20

**Authors:** Shunrong Sun, Wulipan Fulati, Lin Shen, Min Wu, Zilan Huang, Wensi Qian, Pingping Chen, Yingwei Hu, Mingyue Chen, Yu Xu, Hongdi Zhang, Jiexian Ma, Yanhui Xie

**Affiliations:** ^1^ Department of Hematology Huadong Hospital Affiliated with Fudan University Shanghai China

**Keywords:** B‐cell lymphoma, lenalidomide, maintenance therapy, rhGM‐CSF, rituximab

## Abstract

**Background:**

The treatment of high‐risk B‐cell lymphoma (BCL) remains a challenge, especially in the elderly.

**Methods:**

A total of 83 patients (median age 65 years), who have achieved a complete response after induction therapy, were divided into two groups: R^2^ + GM‐CSF regimen (lenalidomide, rituximab, granulocyte‐macrophage colony‐stimulating factor [GM‐CSF]) as maintenance therapy (*n* = 39) and observation (*n* = 44). The efficacy of the R^2^ + GM‐CSF regimen as maintenance in patient with high‐risk BCL was analyzed and compared with observation.

**Results:**

The number of natural killer cells in patients increased after R^2^ + GM‐CSF regimen administration (0.131 × 10^9^/L vs. 0.061 × 10^9^/L, *p* = 0.0244). Patients receiving the R^2^ + GM‐CSF regimen as maintenance therapy had longer remission (duration of response: 18.9 vs. 11.3 months, *p* = 0.001), and longer progression‐free survival (not reached (NR) vs. 31.7 months, *p* = 0.037), and overall survival (OS) (NR vs. NR, *p* = 0.015). The R^2^ + GM‐CSF regimen was safe and well tolerated. High international prognostic index score (*p* = 0.012), and high tumor burden (*p* = 0.005) appeared to be independent prognostic factors for worse PFS.

**Conclusions:**

The maintenance therapy of R^2^ + GM‐CSF regimen may improve survival in high‐risk BCL patients, which might be modulated by amplification of natural killer cells. The efficacy of the R^2^ + GM‐CSF maintenance regimen has to be further validated in prospective random clinical trials.

## 1 INTRODUCTION

High‐risk B‐cell lymphoma (BCL) predominantly affects older individuals, with a median age at diagnosis of 66 years old.^1^ The combination of rituximab, cyclophosphamide, doxorubicin, vincristine, and prednisone (R‐CHOP) administered every 21 days has remained the standard front‐line induction regimen for BCL for over 2 decades; however, the 5‐year survival rates vary from 90% for low‐risk BCL to less than 40%–50% for high‐risk BCL, as defined by the international prognostic index (IPI) score.^2^ Moreover, about 40% of patients eventually fail to achieve complete remission or experience relapse after front‐line rituximab‐chemotherapy.^3^ Older patients with BCL have inferior prognosis due to lower tolerance to conventional chemotherapy and inability to have consolidative autologous stem cell transplantation. There is an urgent need to improve the survival of patients with high‐risk BCL, particularly those who are ineligible for hematopoietic stem cell transplantation (HSCT).

Lenalidomide as maintenance therapy after standard induction chemotherapy showed promising results: It significantly prolonged progression‐free survival (PFS) in older patients with diffuse large B‐cell lymphoma (DLBCL), but failed to improve overall survival (OS).^4^, ^5^ Lenalidomide is an oral immunomodulatory drug that effective against lymphoma. It stimulated T cells and natural killer (NK) cells to migrate into tumor tissue in experimental models.^6^, ^7^, ^8^ Increased NK cell numbers, as well as enhanced function of NK cells, were observed following lenalidomide treatment in malignant BCL, particularly in DLBCL, follicular lymphoma (FL), and mantle cell lymphoma (MCL).^6^ The successful administration of lenalidomide to improve PFS in older patients with DLBCL as maintenance therapy hinted at the importance of modulating the tumor microenvironment.

Tumor microenvironmental cellular components contribute to antitumorigenic functions, survival, and proliferation.^9^ However, NK cells are functionally impaired in patients with high‐risk BCL.^10^ Some studies explored whether BCL inhibits NK cell function by reducing cytoplasmic granulation^11^ and by reducing their ability to form immunological synapses.^12^ Another study proposed that BCL reduced the number of NK cells.^13^ Our research group is attempting to further modify tumor microenvironment based on lenalidomide administration to improve the anti‐tumor effect after induction therapy, increase NK cell number and function, and improve the survival outcome of high‐risk BCL patients, especially the elderly.

It was found that human recombinant granulocyte‐macrophage colony‐stimulating factor (rhGM‐CSF) could increase the number of NK cells.^14^, ^15^ GM‐CSF belongs to a family of hematopoietic cytokines that have a number of autocrine‐mediated effects on immune and non‐immune cells.^16^, ^17^ It increases granulocyte proliferation and phagocytosis, and promotes antibody‐dependent cellular cytotoxicity (ADCC).^16^, ^18^, ^19^ Rituximab is widely used to treat BCL and has synergistic effect with lenalidomide. Lenalidomide treatment enhanced NK cell‐induced cytotoxicity against several rituximab‐treated non‐Hodgkin lymphoma (NHL) cell lines; the effects of lenalidomide‐induced NK cell cytotoxicity and ADCC might be mediated indirectly via interleukin‐2 (IL‐2) production by T cells or Fc‐γ receptors bound to rituximab.^20^, ^21^ We found the combination of rhGM‐CSF, lenalidomide and rituximab (R^2^ + GM‐CSF regimen) could amplify NK cells for about 3 months. We further analyzed the clinical data of R^2^ + GM‐CSF regimen as a maintenance therapy for patients with DLBCL and high‐grade BCL who achieved a complete response (CR), with the ones without any maintenance therapy after induction therapy in our center. We found that the R^2^ + GM‐CSF regimen could prolong the duration of the response and the survival of patients with high‐risk BCL, particularly in the elderly.

## 2 MATERIALS AND METHODS

### 2.1 Patients

Patients were enrolled from January 2013 to November 2021 from the Department of Hematology, Huadong Hospital. Patients received a detailed examination of their tumor tissue (including immunohistochemistry, flow cytometry, fluorescence in situ hybridization, and pathological assessment) using an excisional biopsy specimen evaluated by an expert hematopathologist and was diagnosed CD20^+^ BCL if it met the 2016 WHO diagnostic guidelines for hematopoietic and lymphoid tissue tumors. Following the descriptions of a previous study,^22^, ^23^, ^24^, ^25^, ^26^ we defined high‐risk BCL as: (1) patients with an IPI score of 4–5 (on a scale of 0–5, with higher scores indicating greater risk) in DLBCL; (2) patients with an IPI score <4 but with double‐hit lymphoma (DHL) (*MYC/BCL2, MYC/BCL6*), or triple‐hit lymphoma (THL) (*MYC/BCL‐2/BCL6*), or double‐protein expression (MYC/BCL2), or triple‐protein expression (MYC/BCL2/BCL6), or ABC subtypes with IRF4 or BCL2 hyperexpression; (3) high‐grade BCL with DHL or THL; and (4) high‐grade BCL, NOS. All patients were required to have achieved a CR after induction treatment. Responding patients were assigned to maintenance with the R^2^ + GM‐CSF regimen for 2 years or observation. Patients had no vital organ dysfunction, and their liver, kidney, and heart functions were normal or nearly normal.

This study was approved by the ethical committee of Huadong Hospital (approval number: 2021 K186) and all patients signed informed consent forms.

### 2.2 Treatment

#### 2.2.1 Induction treatment

Patients received an induction chemotherapeutic regimen recommended by the National Comprehensive Cancer Network (NCCN) guidelines based on pathological classification, staging, and general conditions. The dose of chemotherapy drugs and/or the dosing cycle were adjusted according to the side effects of treatment and patient tolerance. The first‐line chemotherapy for BCL is R‐CHOP (rituximab, cyclophosphamide, doxorubicin, vincristine, and prednisone). If patients could not achieve CR after 3 cycles of R‐CHOP chemotherapy, they received second‐line therapy, RICE (rituximab, ifosfamide, cisplatin, and etoposide) with a modified dose for another 6 cycles for the fit elderly who were ineligible for autologous stem cell transplantation.

#### 2.2.2 Maintenance treatment

**FIGURE 1:**
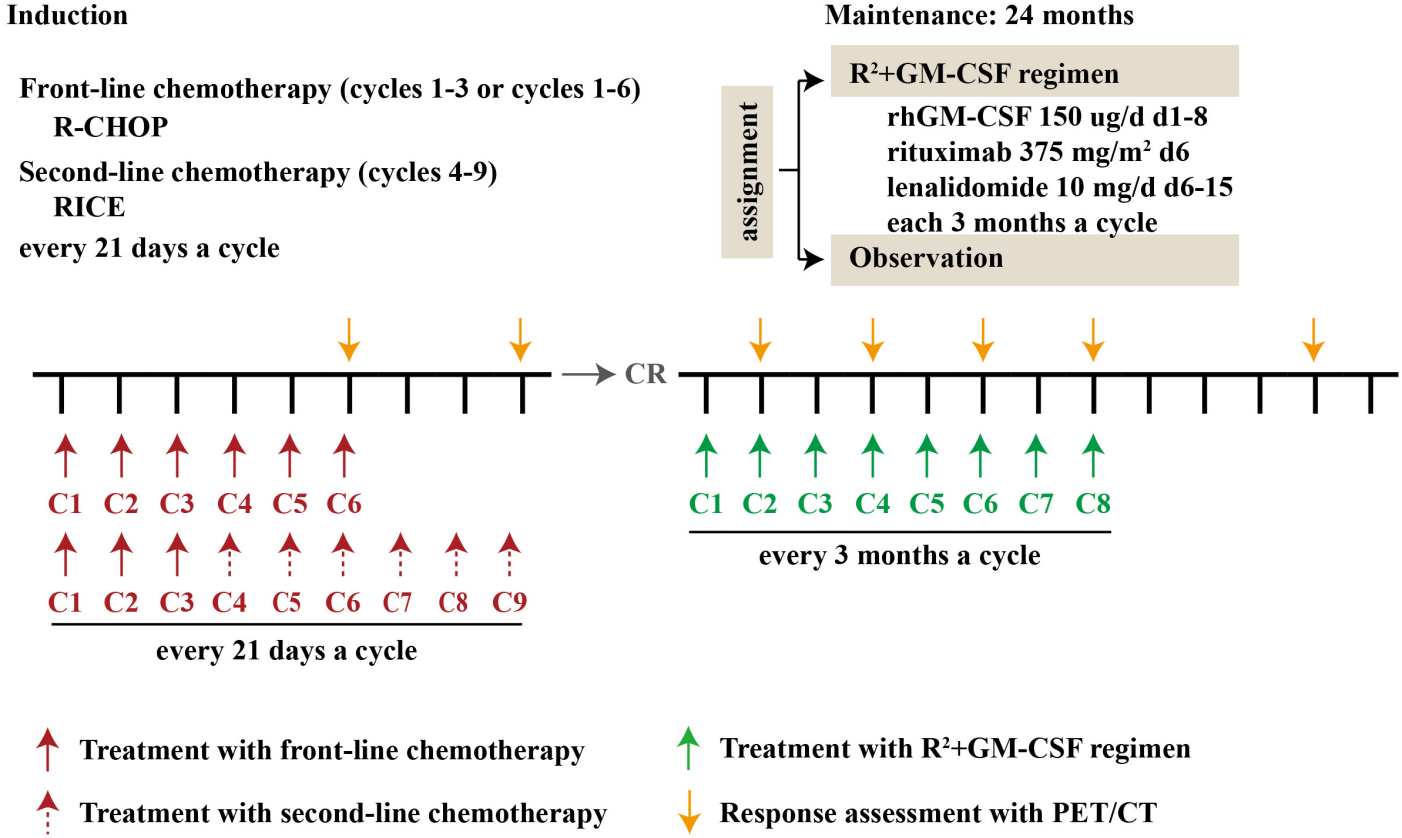
The study design. Patients received 6 or 8 cycles of induction therapy; those responding to induction with a complete response (CR) were enrolled in the study. Patients in the R2 + GM‐CSF group received 24‐month maintenance with R^2^ + GM‐CSF regimen. RhGM‐CSF was administered at a dose of 150 μg/d on Days 1–8; rituximab was administered at a dose of 375 mg/m^2^ on Day 6; and lenalidomide was administered at dose of 10 mg/d on Day 6–15 of each 3‐month‐cycle. The red and green arrows signify treatment with the study drug; the gold arrow signifies response assessments with positron emission tomography‐computerized tomography (PET) scan. Cycle is abbreviated as C.

Lenalidomide, rituximab and rhGM‐CSF have all been approved for the treatment of BCL patients. The observation of patients who achieved CR following induction therapy from 2013 to 2017 did not receive any maintenance therapy, while the R2 + GM‐CSF group of patients from 2018 to 2021 did. The R^2^ + GM‐CSF regimen (rhGM‐CSF (Amoytop Biotech Cop., Xiamen, China) 150 μg/day, subcutaneously on Days 1–8; lenalidomide, 10 mg/day, orally on Days 6–15; rituximab 375 mg/m^2^, intravenous injection on Day 6) was administered every 3 months after induction chemotherapy until completion of 8‐cycle‐maintenance treatment, disease progression or relapse, unacceptable toxicity, or patient refusal (Figure 1). The dosage was adjusted for toxicity. Patients in the observation group stopped receiving any drugs for maintenance after reaching remission under induction chemotherapy. Patients in maintenance therapy group enrolled into this study completed maintenance therapy for at least 4 cycles and all patients were treated as the flowchart shown in Figure S1.

### 2.3 PBMC collection and flow cytometry analysis

Blood was collected from patients in maintenance therapy group. Peripheral blood mononuclear cells (PBMCs) and immune cells were analyzed before to and following each cycle of the maintenance therapy, and then the difference in white blood cells (WBCs), lymphocytes, monocytes, B cells, T cells, and NK cells were compared. Human peripheral blood immune cells were analyzed by Coulter Flow Cytometer (Beckman Coulter, Indianapolis, IN, USA). CD45^+^CD3^+^ is the T‐cell population, CD45^+^CD3^−^CD19^+^ is the B‐cell population and the cell population of CD3^−^CD56^+^/16^+^ is NK cells. The detailed protocol^27^ and materials for flow cytometry are shown in Supplemental materials.

### 2.4 Evaluation of response

Response assessment was analyzed every 3 cycles after induction chemotherapy using a whole body computed tomography (CT) scan, and every 6 cycles using a positron emission tomography‐computerized tomography (PET) scan.

During maintenance, tumor response assessment was performed clinically every 3 months in the first year, every 6 months in the second year, and once a year after the third year using contrast‐enhanced CT scans, ultrasound or PET‐CT. According to the 2016 NCCN recommended LUGANO evaluation criteria, the efficacy evaluation is divided into CR, partial response (PR), stable disease (SD), and progressive disease (PD).

### 2.5 Endpoint and follow‐up

The follow‐up period of this study ended on November 30, 2021. We recorded the data based on the last hospitalization record, out‐patient review, and telephone follow‐up. The duration of response (DOR) was defined as the time from the first evaluation of efficacy as CR or PR to the first evaluation of PD or death from any cause. OS was defined as the time from the date of diagnosis to the date of death of the patient from any cause or the date of the last follow‐up. Progression‐free survival (PFS) was defined as the time from the date of diagnosis to the date of disease progression/relapse, death, or last follow‐up of the patient. In this study, OS and PFS were calculated on a monthly basis.

### 2.6 Evaluation of toxicity

All adverse events (AEs) and serious adverse events (SAEs) reported by the patient or observed by the clinical investigator were collected. An AE was defined as any adverse change from the patient's baseline condition, whether it was considered related to treatment or not. All AEs and SAEs were graded according to the common terminology criteria for adverse events (CTCAE‐version 4.0).

### 2.7 Statistical analysis

The patients' characteristics were presented as mean and standard deviation for numerical variables and as frequencies with percentages for categorical variables. Fisher's exact test for nominal variables and Student's *t*‐test for continuous variables were used to compare the characteristics of the two groups. The Kaplan–Meier method was used to estimate unadjusted probabilities of OS and PFS. The log‐rank test verified the statistical differences between the two groups. Cox regression and logistic regression models were used to obtain HR estimates and corresponding 95% confidence interval (CI) for OS, PFS and DOR. Univariate analyses were performed using Cox proportional hazards regression models to assess for significant predictive risk factors for PFS and OS. SPSS 20.0 software (IBM Corp.) was used to perform all the statistical analyses. A value of *p* < 0.05 was considered statistically significant.

## 3 RESULTS

### 3.1 Patient characteristics

A total of 83 patients who reached CR during induction therapy were included in the study. Among them, 62 patients (32 in the observation group and 30 in the R2 + GM‐CSF group) reached CR after first‐line induction therapy and 21 patients (12 in the observation group and 9 in the R2 + GM‐CSF group) reached CR with second‐line therapy. All patients in the R2 + GM‐CSF group completed at least 4 cycles of maintenance therapy. The baseline characteristics of the patients before receiving maintenance or observation are listed in Table 1. There were no significant differences between the two groups.

**TABLE 1:** Baseline clinical characteristics of patients at diagnosis.

Characteristics	Observation (*n* = 44)	R2 + GM‐CSF (*n* = 39)	χ^2^	*p*
Age, years	64.5 ± 11.76	67.5 ± 11.76	−1.163	0.248
Sex, *n* (%)			0.000	0.987
Male	27 (61.4%)	24 (61.5%)		
Female	17 (38.6%)	15 (38.5%)		
Histology, *n* (%)			5.427	0.246
DLBCL, high IPI (IPI >3)	18 (40.9%)	14 (35.9%)		
DLBCL, DHL or THL (IPI <4)	6 (13.6%)	2 (5.1%)		
DLBCL, DE or TE or ABC subtypes with IRF4 or BCL2 expression (IPI <4)	10 (22.7%)	6 (15.4%)		
High‐grade B‐cell lymphoma, DHL or THL	6 (13.6%)	8 (20.5%)		
High‐grade B‐cell lymphoma, NOS	4 (9.1%)	9 (23.1%)		
Relapse/Refractory lymphoma, *n* (%)	3 (6.8%)	5 (12.8%)	0.855	0.465
Ann Arbor clinical stage at diagnosis, *n* (%)			0.061	0.804
I, II	7 (15.9%)	7 (17.9%)		
III, IV	37 (84.1%)	32 (82.1%)		
IPI score at diagnosis, *n* (%)			2.581	0.275
1–2	17 (38.6%)	11 (28.2%)		
3	9 (20.5%)	14 (35.9%)		
4–5	18 (40.9%)	14 (35.9%)		
ECOG performance status at diagnosis, *n* (%)			4.100	0.060
> 3	10 (22.7%)	17 (43.6%)		
< 3	34 (77.3%)	22 (56.4%)		
Fever, *n* (%)	14 (31.8%)	20 (51.3%)	3.239	0.072
B‐symptoms			0.846	0.358
No	19 (43.2%)	13 (33.3%)		
Yes	25 (56.8%)	26 (66.7%)		
EBV infection, *n* (%)			3.062	0.080
Negative	40 (90.9%)	30 (76.9%)		
Positive	4 (9.1%)	9 (23.1%)		
Bone marrow invasion, *n* (%)			0.219	0.640
Yes	18 (40.9%)	14 (35.9%)		
No	26 (59.1%)	25 (64.1%)		
Metaphase cytogenetics, *n* (%)			0.697	0.404
Normal	38 (86.4%)	31 (79.5%)		
Abnormal	6 (13.6%)	8 (20.5%)		
Elevated LDH (>ULN), *n* (%)			0.049	0.824
No	17 (38.6%)	16 (41.0%)		
Yes	27 (61.4%)	23 (59.0%)		
Elevated beta2‐microglobulin (>ULN), *n* (%)			0.376	0.540
No	23 (52.3%)	23 (59.0%)		
Yes	21 (47.7%)	16 (41.0%)		
Average cycle of induction therapy	6.0 ± 2.18	5.8 ± 1.47	0.386	0.700
Induction therapy			0.192	0.801
First‐line chemotherapy	32 (72.7%)	30 (76.9%)		
Second‐line chemotherapy	12 (27.3%)	9 (23.1%)		

*Note*: Data are presented as no. (%) unless otherwise noted.

Abbreviations: CR, complete response; DLBCL, diffuse large B‐cell lymphoma; EBV, Epstein–Barr virus; ECOG, Eastern Cooperative Oncology Group; IPI, International Prognostic Index; LDH, lactate dehydrogenase; PR, partial response; ULN, upper limit of normal.

### 3.2 The R^2^ + GM‐CSF regimen increased the number of NK cells

**FIGURE 2:**
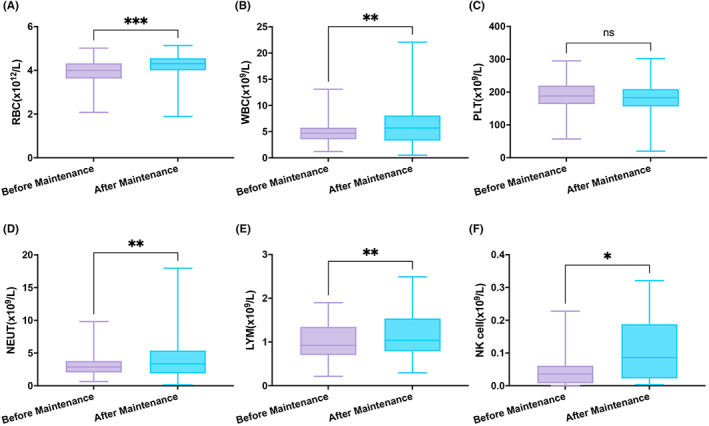
The number of immune cells in peripheral blood before and after R^2^ + GM‐CSF regimen as maintenance therapy in R2 + GM‐CSF group. RBC, red blood cell; WBC, white blood cell; PLT, platelet; NEUT, neutrophil; LYM, lymphocyte. Symbols represent the mean ± SEM and the statistical significance was determined using a paired student *t*‐test or unpaired student *t*‐test with *p* < 0.05 as the threshold for significance. *p < 0.05; **p < 0.01; ***p < 0.001; ns *p* > 0.05.

PBMCs and immune cells were analyzed using flow cytometry before maintenance therapy and after each cycle of maintenance therapy. We observed a statistically significant increase in leukocytes, hemoglobin, and erythrocytes. Figure 2 shows that the number of lymphocytes and NK cells increased significantly after the first cycle of maintenance therapy. The changes of other cells are presented in Figure 2 and supplementary materials.

### 3.3 The R^2^ + GM‐CSF regimen maintained long‐term remission of patients with high‐risk B‐cell lymphoma

The remission status of the patients in the two groups were statistically different. The mean DOR of the 83 patients was 15.0 ± 10.67 months. The DOR in the R2 + GM‐CSF group was longer than that in the observation group (18.8 ± 9.32 months vs. 11.3 ± 10.72 months, *p* = 0.001). The tumor burden was decreased after receiving maintenance therapy (Figure S3).

The difference between the recurrence rates of patients in the observation group and the R2 + GM‐CSF group was 1.6 times greater (20.5% vs. 12.8%), but it was not statistically significant (*p* = 0.242).

### 3.4 The R^2^ + GM‐CSF regimen prolonged the OS of patients with high‐risk B‐cell lymphoma

**FIGURE 3:**
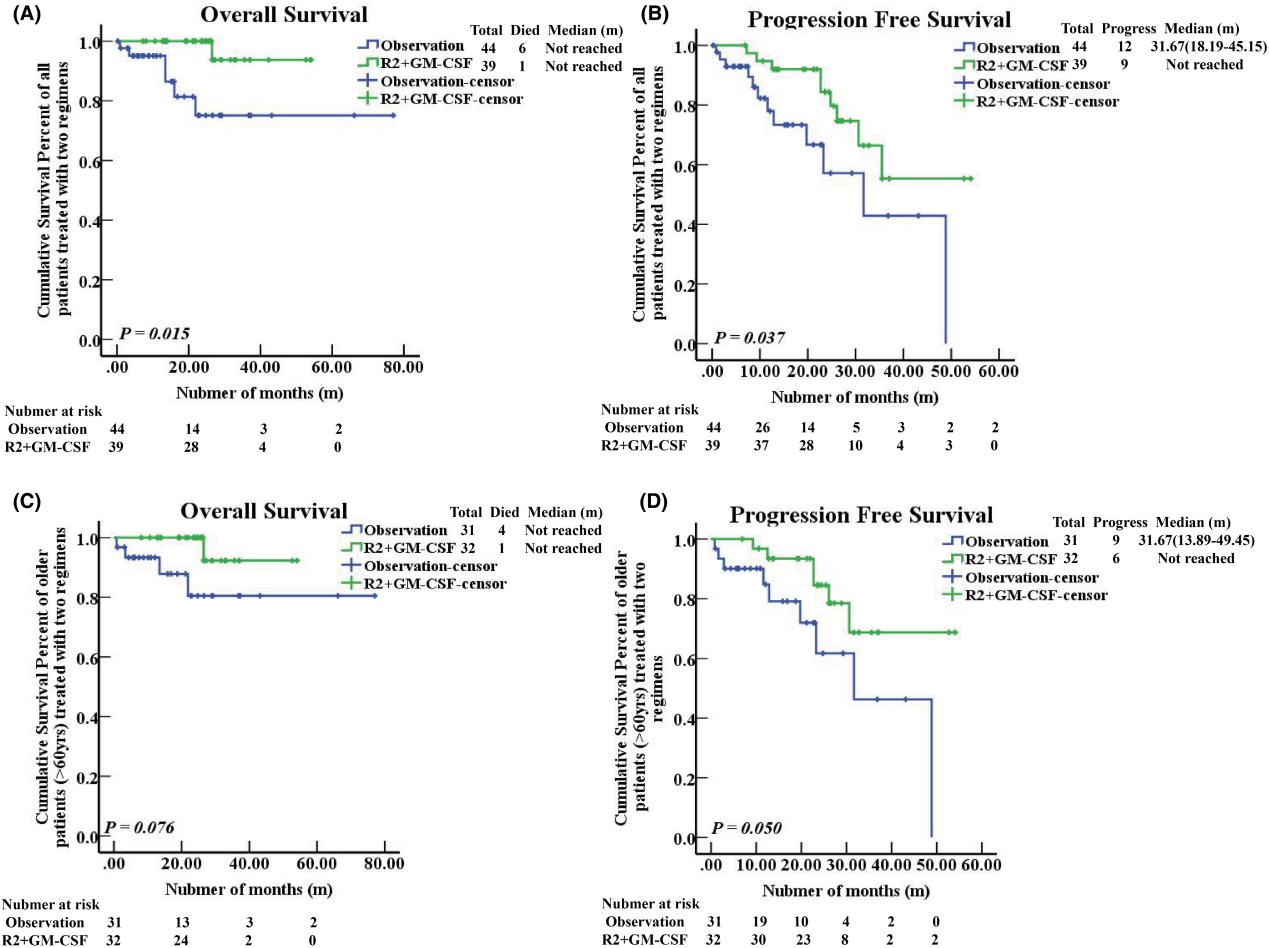
Survival data of patients receiving the R^2^ + GM‐CSF regimen and observation. (A) The overall survival of patients receiving the two regimens (*p* = 0.015). Patients in the R2 + GM‐CSF group and observation group did not reach the median OS time; however, the difference was statistically significant (χ^2^ = 5.924, *p* = 0.015). (B) The progression‐free survival of patients receiving the two regimens (*p* = 0.037). The patients in the R2 + GM‐CSF group did not reach the median PFS time; however, the median PFS time of the patients in the observation group was 31.7 (95% Confidence Interval: 13.89–49.45) months. The difference was statistically significant (χ^2^ = 4.355, *p* = 0.037). (C) The overall survival of patients who were older than 60 years (*p* = 0.076). Patients in the R2 + GM‐CSF group and observation group did not reach the median OS time, but the difference was not statistically significant (χ^2^ = 3.148, *p* = 0.076). (D) The progression‐free survival of patients who were older than 60 years (*p* = 0.050). The patients in the R2 + GM‐CSF group did not reach the median PFS time, but the median PFS time of patients in observation group was 31.7 (95% CI: 18.19–45.15) months. The difference was statistically significant (χ^2^ = 3.836, *p* = 0.050).

At a longer median follow‐up of 21.1 months (range, 0.2–77.0 months), the median OS was not reached in either group; however, the difference in OS between the two groups was statistically significant (hazard ratio (HR) favoring the R^2^ + GM‐CSF regimen, 0.112, 95% CI, 0.013–0.941; *p* = 0.015, Figure 3A). The 1‐year OS rates of the R2 + GM‐CSF group and the observation group were 100% and 95.5%, and there was no significant difference (*p* = 0.165). However, the 5‐year OS rates were 97.4% and 86.4%, respectively, and the difference was statistically significant (*p* = 0.015).

The OS benefit of the R^2^ + GM‐CSF regimen over observation showed a tendency among older patients (age above 60) with high‐risk BCL (*p* = 0.076, Figure 3C). In these patients, the 1‐year OS rates of the R2 + GM‐CSF and observation groups were 100% and 93.5%, and the 5‐year OS rates were 96.9% and 87.1%; however, the difference was not statistically significant (*p* = 0.238, *p* = 0.196).

### 3.5 The R^2^ + GM‐CSF regimen prolonged PFS of patients with high‐risk B‐cell lymphoma

With a median follow‐up of 18.4 months (range, 0.2–54.1 months), the R^2^ + GM‐CSF regimen as maintenance treatment enabled patients to achieve longer PFS. The median PFS was not reached in the R2 + GM‐CSF group, but was estimated at 31.7 months in the observation group (HR favoring the R^2^ + GM‐CSF regimen, 0.404, 95% CI, 0.168–0.973; *p* = 0.037, Figure 3B). The 1‐year PFS rates and 5‐year PFS rates of the R2 + GM‐CSF group and observation group were 100% and 84.1%, and 76.9%, and 72.7%, respectively, and the differences were statistically significant (*p* = 0.003, *p* = 0.037).

The PFS benefit of the R^2^ + GM‐CSF regimen over observation was likely to be seen among older patients with high‐risk BCL (HR favoring R^2^ + GM‐CSF regimen, 0.368, 95% CI, 0.129–1.000; *p* = 0.050, Figure 3D). In these patients, the 1‐year PFS rates and 5‐year PFS rates of the R2 + GM‐CSF group and observation group were 100% and 87.1%, and 81.2% and 71%, respectively; however, the differences were not statistically significant (*p* = 0.053, *p* = 0.387).

### 3.6 Safety

Safety during maintenance therapy was assessed for 39 patients who undertook at least 1 cycle. We collected all of immediate responses to analyze the side effects. All AEs are shown in Table 2. Hematological toxicities that occurred more frequently in the R2 + GM‐CSF group included anemia (13/39), lymphocytopenia (9/39), and leukopenia (9/39). All of the hematological adverse events were Grades 1 and 2 (16/39), and none were Grade 3 or 4. Besides, common treatment‐related non‐hematological adverse events were of Grades 1 and 2 (16/20), with the most frequent ones being nausea or vomiting (6/39). Only one patient had a fever as a result of the rhGM‐CSF. Notably, only one patient died (1/39) during the study. In short, the side effects were so mild that none of them required management through dose interruptions and/or reductions and other support, and no serious or fatal adverse events (Grade above 3) occurred.

**TABLE 2:** Treatment‐emergent adverse events in patients receiving R2 + GM‐CSF.

Adverse events^a^	*N* (*n* = 39)	
	Grades 1 and 2	Grade 3 or 4
Hematological adverse events	23	0
Anemia	13	0
Leukopenia	9	0
Neutropenia	2	0
Lymphocytopenia	9	0
Thrombocytopenia	2	0
Non‐hematological adverse events	6	0
Constipation	1	0
Cough	3	0
Fatigue	1	0
Pyrexia	3	0
Upper respiratory tract infection	2	0
Abdominal pain	1	0
Nausea or vomiting	6	0
Rash	1	0
Alanine aminotransferase increased	3	0
Back pain	2	0

*Note*: Data are presented as no. (%) unless otherwise noted.

^a^ Safety during maintenance was assessed for patients who undertook at least one visit. All adverse events, defined as any adverse change from the patient's baseline condition, whether considered related to treatment or not, were collected and graded according to the common terminology criteria for adverse events 4.0 grading system.

### 3.7 Prognostic factors

**FIGURE 4:**
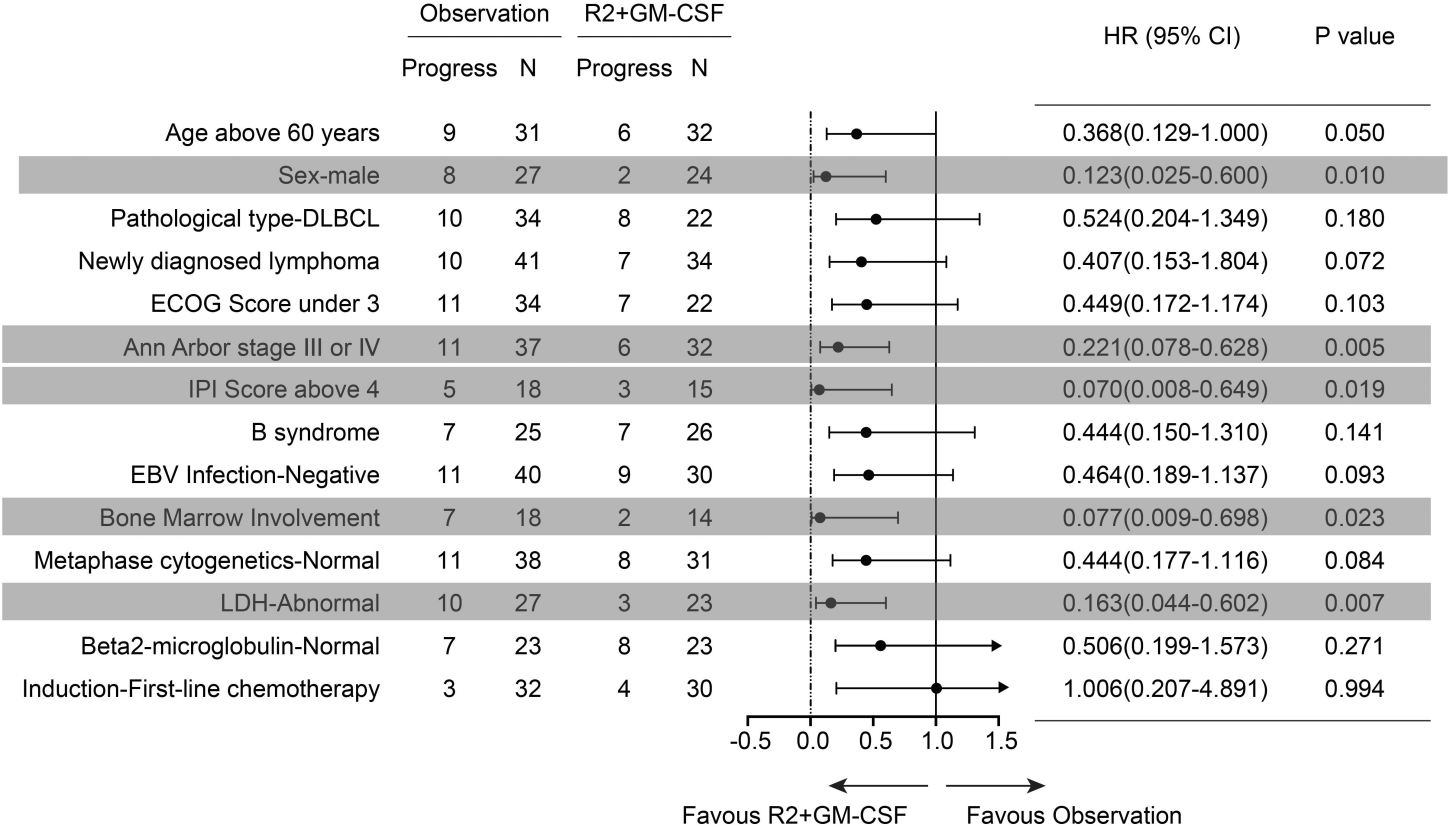
Subgroup analyses of progression‐free survival. Meta‐analysis (Forest) plot showing the results of subgroup analysis of progression‐free survival among 83 patients. The dashed vertical line indicates a hazard ratio of 0, and the solid vertical line indicates a hazard ratio of 1.0.

For patients who were older than 60 years old, the R^2^ + GM‐CSF regimen as maintenance therapy seem to be helpful to prolong their PFS. If those patients discontinue to receive the corresponding treatment after induction of remission, there would be an increased risk of disease progression, as evidenced by a shorter PFS time, although there was no significant difference [*p* = 0.050, HR 0.368 (95% CI 0.129–1.000), Figure 4].

Additionally, univariate analysis found that compared with the observation group, the R^2^ + GM‐CSF regimen as maintenance therapy was helpful in prolonging the long‐term PFS rate, especially in patients with advanced‐staged (III or IV) disease, patients with an IPI score greater than four, and patients with bone marrow invasion. The details are presented in Figure 4 and the Table S2.

## 4 DISCUSSION

Prior studied have established maintenance therapy, which includes rituximab monotherapy,^28^, ^29^, ^30^ rituximab in combination with lenalidomide,^31^ and obinutuzuamb plus bendamustine,^32^ as a successful strategy to prolong the PFS and OS of patients in some MCL and FL clinical settings; however, few studies have shown survival benefit of maintenance therapy in high‐risk BCL. Lenalidomide maintenance therapy was first shown in phase II multicenter studies to improve PFS in high‐risk patients with relapsed DLBCL who were ineligible for stem cell transplantation.^33^, ^34^ In addition, the phase III REMARC trial showed that there was no superiority in prolonging OS (lenalidomide vs. placebo) in elderly patients following front‐line R‐CHOP induction.^4^ The present cohort study focused on modifying the maintenance therapy and enhancing the effect of lenalidomide to kill residual tumor cells. Lenalidomide, rituximab, and rhGM‐CSF were all approved for the treatment of BCL, then they were combined as a maintenance therapy in our study. Lenalidomide is an oral immunomodulator with direct anti‐tumor activity and indirect anti‐neoplastic actions, which occur through recruiting NK cells to tumor sites and stimulating the proliferation and activation of NK cells (and other immune cells) to modify the tumor microenvironment.^35^ In addition, rhGM‐CSF can increase the numbers and cytotoxicity of effector cells,^36^, ^37^, ^38^ and the biological effect combined with rituximab might enhance patients' innate immune response against NHL.^16^ Firstly, we found that the R^2^ + GM‐CSF regimen amplify NK cell numbers. Secondly, following induction chemoimmunotherapy, the R^2^ + GM‐CSF regimen as maintenance therapy significantly prolonged PFS and OS compared to observation, and delayed the time to recurrence. Thirdly, the R^2^ + GM‐CSF regimen was well tolerated, even in elderly patients. The R^2^ + GM‐CSF regimen seemed to improve the PFS and showed a tendency to improve OS in elderly high‐risk, high tumor burden patients; therefore, the R^2^ + GM‐CSF regimen might offer a novel choice for these patients.

The strategy of R^2^ + GM‐CSF regimen might take advantage of this potential effect to amplify the number of NK cells as well as the potential enhancement of NK function in high‐risk BCL, which might eradicate residual lymphoma cells, avoiding both early and late relapse. Given the greater number of NK cells in maintenance group compared to the observation group in our small cohort trail, it is likely that the clinical benefit observed from R^2^ + GM‐CSF regimen maintenance therapy could be due to an immunomodulatory mechanism.

Lenalidomide have been shown to have toxicities, with Grade 3 and 4 neutropenia (57%) and fatigue (13%)^4^, ^33^, ^39^ occurring more frequently and leading to more premature discontinuations. Due to the addition of rhGM‐CSF, the toxicities in current research, however, were not as expected. The addition of rhGM‐CSF might shield NHL patients against neutropenia and febrile neutropenia. The R^2^ + GM‐CSF regimen appears to be more tolerable for older patients.

This study showed that maintenance treatment using the R^2^ + GM‐CSF regimen may have a survival advantage for patients with high‐risk BCL. A limitation of this study was the small number of patients and the retrospective study itself. We found that the R^2^ + GM‐CSF regimen improved the OS and PFS in high‐risk BCL; however, the possibility of tumor microenvironment modulation in treatment of older BCL patients seems to be the highlight of this study. The conclusions should be validated in prospective, randomized, multicenter clinical trials.

## AUTHOR CONTRIBUTIONS

**Shunrong Sun:** Data curation (equal); formal analysis (equal); investigation (equal); writing – original draft (equal); writing – review and editing (equal). **Wulipan Fulata:** Data curation (equal); formal analysis (equal); writing – review and editing (equal). **Lin Shen:** Conceptualization (equal); funding acquisition (equal); project administration (equal); supervision (supporting). **Min Wu:** Data curation (equal); investigation (equal); project administration (equal); supervision (equal). **Zilang Huang:** Project administration (equal); resources (equal); software (equal). **Wensi Qian:** Project administration (equal); supervision (equal). **Pingping Chen:** Investigation (equal); project administration (equal). **Yingwei Hu:** Investigation (equal); project administration (equal). **MIngyue Chen:** Investigation (equal); project administration (equal). **Yu Xu:** Investigation (equal); project administration (equal). **Hongdi Zhang:** Conceptualization (equal); investigation (equal); methodology (equal); project administration (equal). **Jiexian Ma:** Conceptualization (equal); formal analysis (equal); funding acquisition (lead); investigation (equal); project administration (equal); supervision (equal); visualization (equal); writing – review and editing (equal). **Yanhui Xie:** Conceptualization (lead); methodology (lead); project administration (lead); supervision (supporting); writing – review and editing (supporting).

## FUNDING INFORMATION

This work was supported by the Shanghai Science and Technology Committee [grant numbers 21Y11909000 and 19DZ1910300], the Shanghai Health Committee [grant number 202140518], the Elite Project of Huadong Hospital (HD1439), and the Project of Huadong Hospital (20191c014).

## CONFLICT OF INTEREST STATEMENT

No financial or other conflicts of interest to disclose.

## ETHICS STATEMENT

The study protocols were approved by the ethical committee of Huadong Hospital (approval number: 2021 K186). The patients provided written informed consent. This clinical investigation was conducted according to the principles of the Declaration of Helsinki.

## Supporting information


Data S1.

## Data Availability

The datasets used and/or analyzed during the current study are available from the corresponding author on reasonable request.
