# Supplementary material for: Maintenance regimen of GM‐CSF with rituximab and lenalidomide improves survival in high‐risk B‐cell lymphoma by modulating natural killer cells
Source: Cancer Med. 2023 Apr 20;12(12):12975–85. doi: 10.1002/cam4.5969 (PMC10315792; doi:10.1002/cam4.5969)
Supplement: Supplementary file 1 — Data S1. [file CAM4-12-12975-s001.docx]

Supplement material

Methods

Flow Cytometers Analysis

Eight different surface markers were used in the flow cytometry studies. List mode reanalysis was performed using gating for lymphocytes, monocytes, and myeloid populations, based on side scatter-CD45 histograms ^1^. The monoclonal antibodies include isotype controls, and those recognizing CD45, CD3, CD4, CD8, CD19, CD56/16, CD5, and CD7 (BD Horizon™, BD Biosciences, San Jose, CA, USA). In this experiment, human peripheral blood immune cells were mainly divided into lymphocyte and monocyte groups according to cell size and the granularity they contained, namely FSC and SSC. Then, we observed the expression of CD45, CD3, and CD19, among which CD45^+^CD3^+^ is the T cell population, and CD45^+^CD3^-^CD19^+^ is the B cell population. To subdivide T cells, T cells can be divided into helper T cells (CD45^+^CD3^+^CD4^+^) and killer T cells (CD45^+^CD3^+^CD8^+^) by CD4 and CD8. The cell population of CD3^-^CD56^+^/16^+^ is NK cells. The samples were analyzed on a Coulter Flow Cytometer (Beckman Coulter, Indianapolis, IN, USA) equipped with a 488 nm Argon laser. The results were reanalyzed using FlowJo™ software (FlowJO LLC, Ashland, OR, USA). Flow cytometry data were compared with patient characteristics, including blood counts ^1^.

Supplementary Table 1. Changes in the numbers of immune cells before and after R2+GM-CSF maintenance therapy

|  | Before R2+GM-CSF | After R2+GM-CSF | t | *P* value |
| --- | --- | --- | --- | --- |
| RBC (×10^12^/L) | 3.96 | 4.18 | 4.082 | 0.0002 |
| HGB (g/L) | 119.08 | 126.36 | 4.581 | < 0.001 |
| PLT (×10^9^/L) | 190.42 | 181.46 | 1.341 | 0.1863 |
| WBC (×10^9^/L) | 4.85 | 6.46 | 2.951 | 0.0048 |
| NEUT (×10^9^/L) | 3.13 | 4.49 | 2.806 | 0.0072 |
| LYM (×10^9^/L) | 1.03 | 1.19 | 2.862 | 0.0062 |
| MONO (×10^9^/L) | 0.47 | 0.52 | 0.8458 | 0.4018 |
| EOS (×10^9^/L) | 0.19 | 0.23 | 1.087 | 0.2823 |
| BASO (×10^9^/L) | 0.0281 | 0.0283 | 0.04485 | 0.9644 |

Note. Data are presented as No. (%) unless otherwise noted.

Abbreviations: RBC, red blood cell; HGB, hemoglobin; PLT, platelet; WBC, white blood cell; NEUT, neutrophil; LYM, lymphocyte; MONO, monocyte; ESO, basophilic granulocyte; BASO: eosinophilic granulocyte.

Supplement Table 2 Comparison of OS and PFS Associated with the Two regimens (Observation and R2+GM-CSF) Stratified by Baseline Characteristics

| Baseline Characteristics |  | *P* value | HR (95%CI) |
| --- | --- | --- | --- |
| Age under 60 years  (R2 vs Without R2) | OS  PFS | 0.439  0.312 | 0.010(0-1239.594)  0.310(0.032-3.007) |
| Age above 60 years  (R2 vs Without R2) | OS  PFS | 0.116  0.050 | 0.172(0.019-1.549)  0.368(0.129-1.000) |
| Sex-male | OS  PFS | 0.284  0.010 | 0.012(0-40.519)  0.123(0.025-0.600) |
| Sex-female | OS  PFS | 0.237  0.491 | 0.233(0.021-2.607)  1.622(0.410-6.426) |
| Pathological type-DLBCL | OS  PFS | 0.095  0.180 | 0.158(0.018-1.374)  0.524(0.204-1.349) |
| Pathological type-B-cell lymphoma, unclassifiable | OS  PFS | 0.582  0.462 | 0.006(0-500061.287)  0.003(0-16320.399) |
| Relapse/Refractory lymphoma | OS  PFS | 0.436  0.436 | 0.005(0-3577.948)  0.005(0-3577.948) |
| Newly diagnosed lymphoma | OS  PFS | 0.114  0.072 | 0.170(0.019-1.532)  0.407(0.153-1.084) |
| ECOG Score under 3 | OS  PFS | 0.129  0.103 | 0.189(0.022-1.627)  0.449(0.172-1.174) |
| ECOG Score above 3 | OS  PFS | 0.929  0.208 | .*  0.157(0.009-2.797) |
| Ann Arbor stage I or II | OS  PFS | 0.584  0.408 | 105.972(0-1909925145)  3.244(0.004-650933.934) |
| Ann Arbor stage III or IV | OS  PFS | 0.179  0.005 | 0.009(0-8.567)  0.221(0.078-0.628) |
| IPI Score under 4 | OS  PFS | 0.308  0.951 | 0.287(0.026-3.613)  1.039(0.310-3.583) |
| IPI Score above 4 | OS  PFS | 0.303  0.019 | 0.014(0-46.317)  0.070(0.008-0.649) |
| B syndrome | OS  PFS | 0.268  0.141 | 0.008(0-41.849)  0.444(0.150-1.310) |
| Not B syndrome | OS  PFS | 0.686  0.235 | 0.609(0.055-6.758)  0.364(0.068-1.933) |
| EBV Infection positive | OS  PFS | 0.586  0.586 | 0.005(0-1150000.899)  0.005(0-1150000.899) |
| EBV Infection negative | OS  PFS | 0.083  0.093 | 0.149(0.017-1.283)  0.464(0.189-1.137) |
| Bone Marrow Involvement | OS  PFS | 0.309  0.023 | 0.015(0-48.510)  0.077(0.009-0.698) |
| Without Bone Marrow Involvement | OS  PFS | 0.286  0.756 | 0.271(0.024-2.988)  0.832(0.261-2.651) |
| Metaphase cytogenetics-Normal | OS  PFS | 0.080  0.084 | 0.147(0.017-1.262)  0.444(0.177-1.116) |
| Metaphase cytogenetics-Abnormal | OS  PFS | 0.590  0.493 | 0.011(0-143420.831)  0.354(0.018-1.892) |
| LDH- Normal | OS  PFS | 0.667  0.578 | .  1.582(0.044-0.602) |
| LDH- Abnormal | OS  PFS | 0.183  0.007 | 0.01(0-8.707)  0.163(0.044-0.602) |
| Beta2-microglobulin- Normal | OS  PFS | 0.166  0.271 | 0.200(0.021-1.945)  0.560(0.199-1.573) |
| Beta2-microglobulin- Abnormal | OS  PFS | 0.368  0.047 | 0.014(0-156.534)  0.111(0.013-0.975) |
| Induction-First-line chemotherapy | OS  PFS | 0.373  0.994 | 0.260(0.013-5.031)  1.006(0.207-4.891) |
| Induction-Non-first-line chemotherapy | OS  PFS | 0.484  0.363 | 0.035(0-424.128)  0.382(0.048-3.040) |

*. Unable to calculate statistics

Supplementary Table 3 The information of antibody

|  | antibody | Clone | Cat# | Vol. Per Test |
| --- | --- | --- | --- | --- |
| CD45 | BD Horizon^TM^ V500 mouse Anti-Human CD45 | HI30 | 560777 | 5μL |
| CD3 | BD^TM^ PE-Cy^TM^7 Mouse Anti-Human CD3 | SK7 | 341091 | 5μL |
| CD4 | BD^TM^ CD4 APC-Cy^TM^7 | SK3 | 341115 | 5μL |
| CD8 | BD^TM^ APC Mouse Anti-Human CD8 | SK1 | 340584 | 5μL |
| CD19 | BD^TM^ PerCP-CyTM5.5 Mouse Anti-Human CD19 | SJ25C1 | 340951 | 20μL |
| CD56 | BD^TM^ FITC Mouse Anti-Human CD56 | NCAM16.2 | 340410 | 20μL |
| CD16 | BD^TM^ CD16 FITC | NKP15 | 335035 | 20μL |
| CD7 | BD^TM^ PE Mouse Anti-Human CD7 | M-T701 | 340581 | 20μL |
| CD5 | BD Horizon^TM^ V450 Mouse Anti-Human CD5 | L17F12 | 644487 | 5μL |

Supplementary Figure 1. The flowchart of treatment regiments. Patients received six or eight cycles of induction therapy; those responding to induction with a complete response (CR) were enrolled in the study. Patients in the R2+GM-CSF group received 24-month maintenance with new R^2^ regimen.


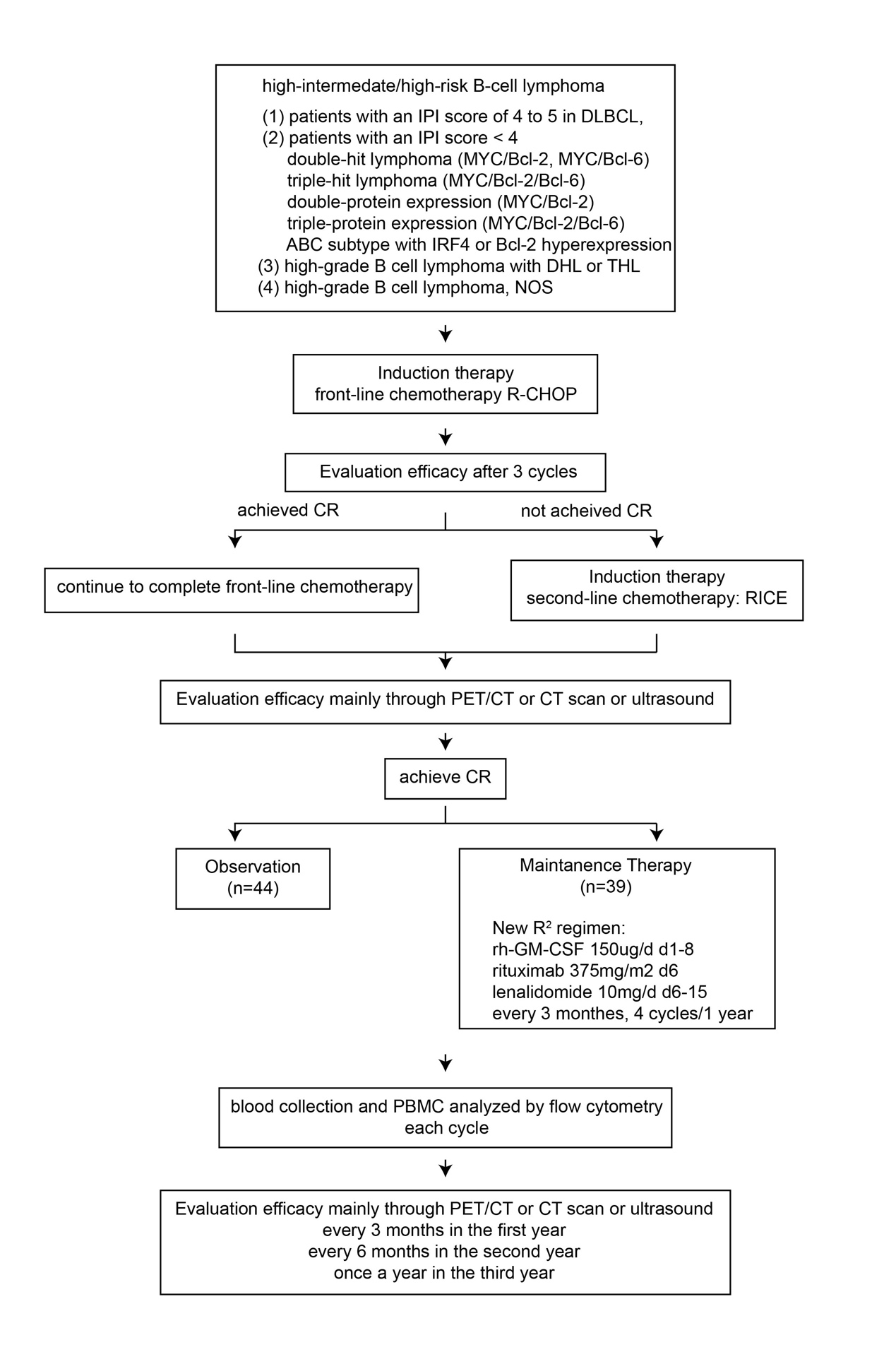


Supplement Figure 2 Changes in the numbers of other PBMCs and immune cells before and after R2+GM-CSF maintenance therapy in R2+GM-CSF group. Symbols represent the mean ± SEM and the statistical significance was determined using a paired student *t* test or unpaired student *t* test with *P* < 0.05 as the threshold for significance. **P* < 0.05; *****P* < 0.0001. HGB, hemoglobin; PLT, platelet; MONO, monocyte; ESO, basophilic granulocyte; BASO: eosinophilic granulocyte.


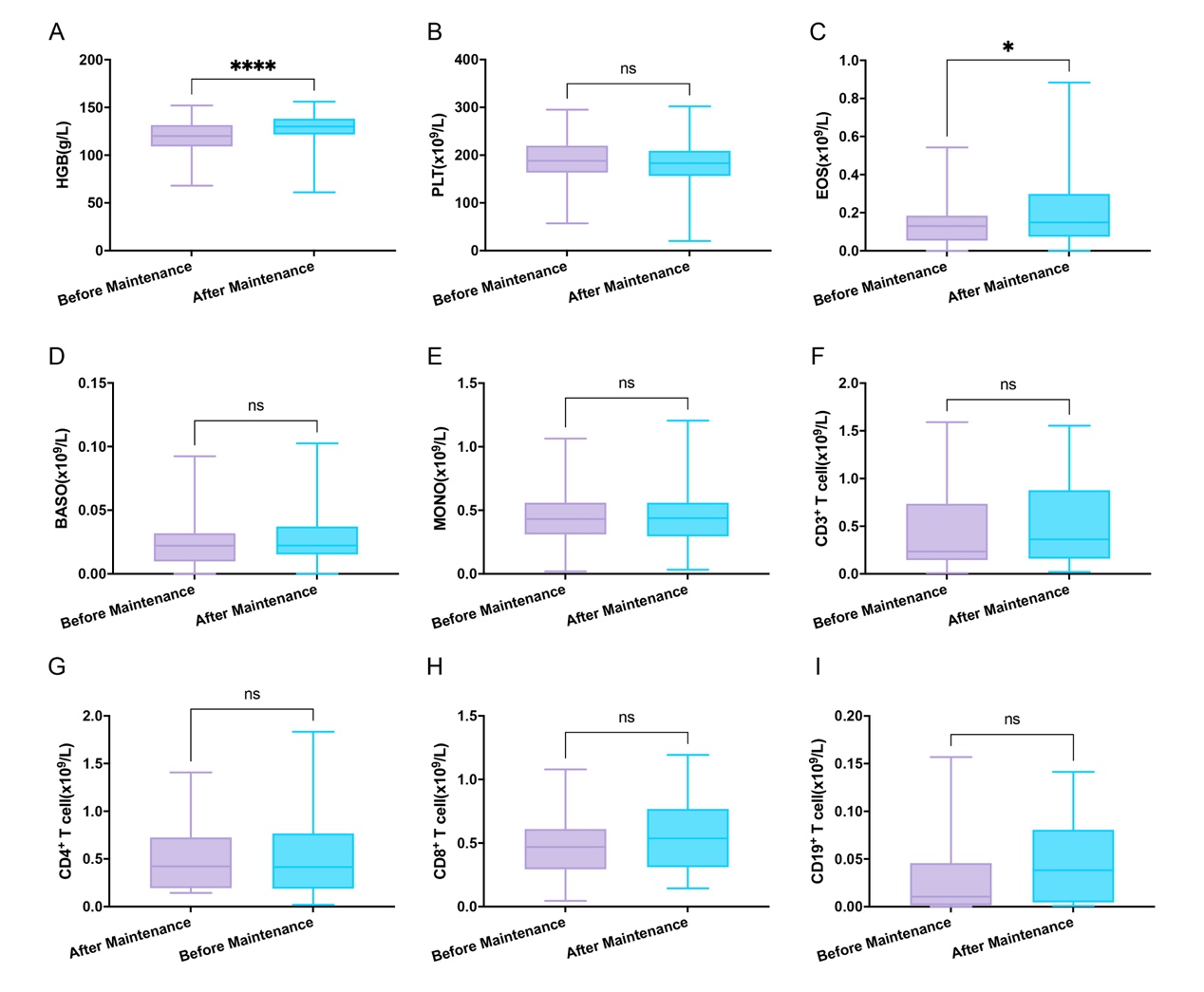


Supplement Figure 3 Comparative PET-CT image of a patients in R2+GM-CSF group at the diagnosis of the disease, before maintenance therapy and after maintenance therapy.


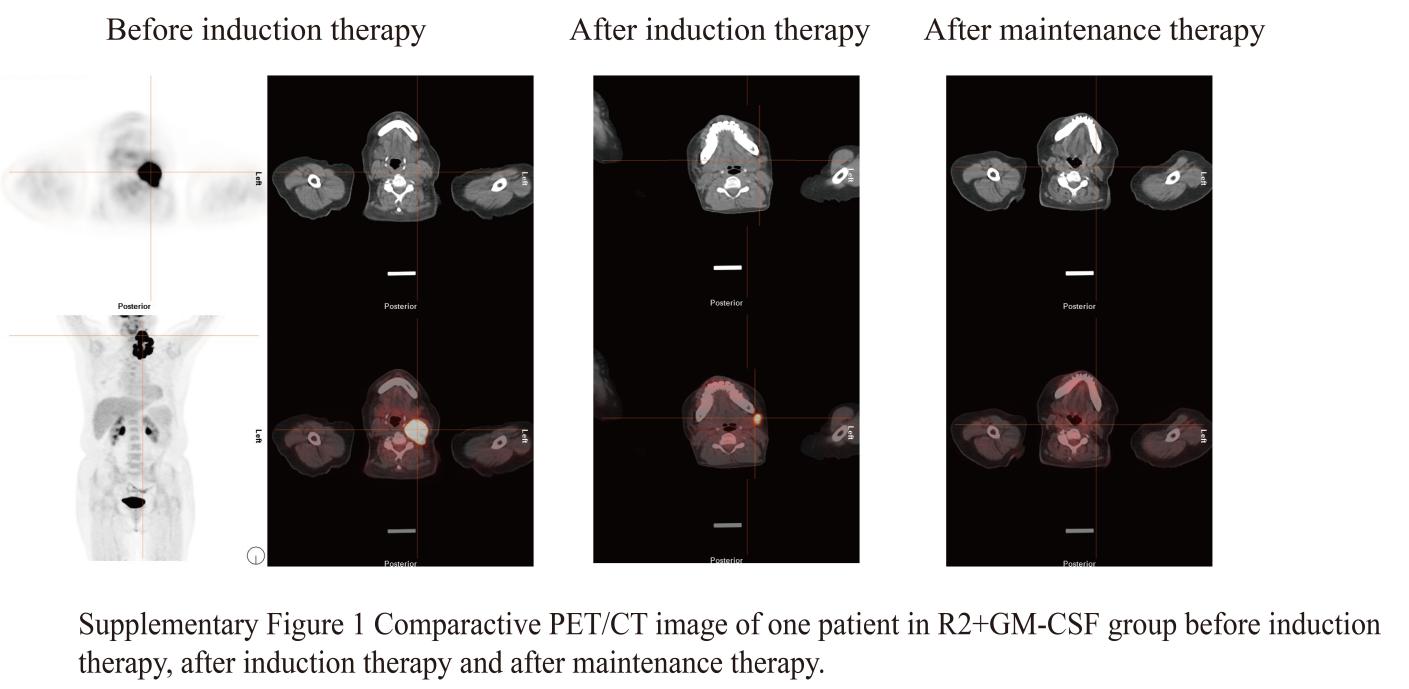


Reference

1. Savaşan S, AlQanber B, Henry M, Buck S, Gadgeel M. Differing reflections of paediatric classical Hodgkin's lymphoma on local and distant immunological microenvironments: a flow cytometric study. *J Clin Pathol.* 2020;73(3):176-179.
